# Liquid Biopsies for Monitoring Medulloblastoma: Circulating Tumor DNA as a Biomarker for Disease Progression and Treatment Response

**DOI:** 10.7759/cureus.51712

**Published:** 2024-01-05

**Authors:** Barbara Buccilli, Maria A Rodriguez Molina, Daniela P Redrovan Palomeque, Cindy A Herrera Sabán, Fides M C. Caliwag, Cristian Jenifer S Contreras Flores, Chamathi W. J Abeysiriwardana, Edna Diarte, Victor S Arruarana, Ernesto Calderon Martinez

**Affiliations:** 1 Department of Human Neuroscience, Sapienza University of Rome, Rome, ITA; 2 Department of Neurosurgery, Mount Sinai Hospital, New York, USA; 3 Department of Pediatrics, Facultad de Medicina de la Universidad de Panamá, Panamá, PAN; 4 Department of General Practice, University de la Cuenca, Quito, ECU; 5 Department of General Practice, Facultad de Ciencias Médicas, Universidad de San Carlos de Guatemala, San Carlos, GTM; 6 Department of General Practice, Ateneo School of Medicine and Public Health, Pasig City, PHL; 7 Department of Internal Medicine, St. George's University School of Medicine, Grenada, USA; 8 Department of General Practice, Caribbean Medical University School of Medicine, Willemstad, CUW; 9 Department of Medicine, Universidad Autónoma de Sinaloa, Culiacán, MEX; 10 Department of Internal Medicine, Brookdale University Hospital Medical Center, New York, USA; 11 Department of Digital Health, Universidad Nacional Autónoma de México, Mexico City, MEX

**Keywords:** wnt, ssh, medulloblastoma, ctdna, liquid biopsy

## Abstract

Pediatric brain tumors, including medulloblastoma (MB), represent a significant challenge in clinical oncology. Early diagnosis, accurate monitoring of therapeutic response, and the detection of minimal residual disease (MRD) are crucial for improving outcomes in these patients. This review aims to explore recent advancements in liquid biopsy techniques for monitoring pediatric brain tumors, with a specific focus on medulloblastoma. The primary research question is how liquid biopsy techniques can be effectively utilized for these purposes. Liquid biopsies, particularly the analysis of circulating tumor DNA (ctDNA) in cerebrospinal fluid (CSF), are investigated as promising noninvasive tools. This comprehensive review examines the components of liquid biopsies, including ctDNA, cell-free DNA (cfDNA), and microRNA (miRNA). Their applications in diagnosis, prognosis, and MRD assessment are critically assessed. The review also discusses the role of liquid biopsies in categorizing medulloblastoma subgroups, risk stratification, and the identification of therapeutic targets. Liquid biopsies have shown promising applications in the pediatric brain tumor field, particularly in medulloblastoma. They offer noninvasive means of diagnosis, monitoring treatment response, and detecting MRD. These biopsies have played a pivotal role in subgroup classification and risk stratification of medulloblastoma patients, aiding in the identification of therapeutic targets. However, challenges related to sensitivity and specificity are noted. In conclusion, this review highlights the growing importance of liquid biopsies, specifically ctDNA analysis in CSF, in pediatric brain tumor management, with a primary focus on medulloblastoma. Liquid biopsies have the potential to revolutionize patient care by enabling early diagnosis, accurate monitoring, and MRD detection. Nevertheless, further research is essential to validate their clinical utility fully. The evolving landscape of liquid biopsy applications underscores their promise in improving outcomes for pediatric brain tumor patients.

## Introduction and background

As one of the most common malignant pediatric brain tumors, medulloblastoma (MB) [1] has seen many recent advancements, including a shift from histology-based diagnostics to molecular and genetic profiling. This transition aims to establish a stronger correlation between tumor characteristics and clinical outcomes, improving the understanding and management of the disease [2]. The incidence of medulloblastoma is age-dependent [3], showing variation from 0.16 to 0.63 per 100,000 (Table 1).

**Table 1:** Incidence of medulloblastoma by age The incidence is presented as the number of cases per 100,000 people in the general population [3].

Age	Incidence
0-4 years	0.51 per 100,000
5-9 years	0.63 per 100,000
10-14 years	0.33 per 100,000
14-19 years	0.16 per 100,000

The mortality rate is close to one-third [4]. It is still difficult to assess responses using imaging and cerebrospinal fluid (CSF) cytology, and there is no marker for minimal residual disease (MRD) [4]. Approximately 70% of those receiving therapy are successfully treated with a multimodal approach, combining surgery, radiation, and chemotherapy [5-8]. Individuals who experience disease progression typically die from it, while those who survive frequently experience long-term side effects from their treatments [9,10]. This emphasizes the importance of a well-stratified frontline therapy. Patients with resistant tumors might be saved through treatment intensification and prompt second-line or experimental approaches made possible by early identification of recurrence. Invasive neurosurgery interventions are needed to analyze tumor-derived genetic data upon recurrence [9]. To optimize outcomes after failure of the frontline treatment, reliable, noninvasive markers are needed. Even though molecular genetic information has increasingly become a part of diagnostic neuropathology, applying the outcomes of cutting-edge molecular assays to clinical practice is still a challenge [11].

The International Collaboration on Cancer Reporting's recommendations provide a list of tumors' potential immunohistochemical or molecular characteristics, along with a template to report their presence [12,13]. In medulloblastoma, the morphological subtype and the molecular subgroup should be provided with additional molecular data. This shows the clinical relevance of these characteristics. A panel approach to immunohistochemistry may help with subgrouping [11] and integrated histologic and molecular diagnostics with gathering prognostic data and guiding individualized approaches [2].

Spinal cord metastases have long been considered a result of "drop metastasis" into the ventricular system and subsequent transfer via the CSF. Still, a xenograft model has shown recent evidence of hematogenous dissemination [14]. This supports looking for circulating tumor cells (CTCs) in these patients' blood. By tracking biomarker levels, CSF can be employed for both initial diagnosis and to anticipate therapy outcomes and recurrences [15].

This review aims to provide a comprehensive overview of the current state and potential of liquid biopsies using circulating tumor DNA (ctDNA) in the context of medulloblastoma monitoring and open the door to further research to improve these patients' outcomes and quality of life.

## Review

WNT-activated medulloblastoma

**Figure 1:**
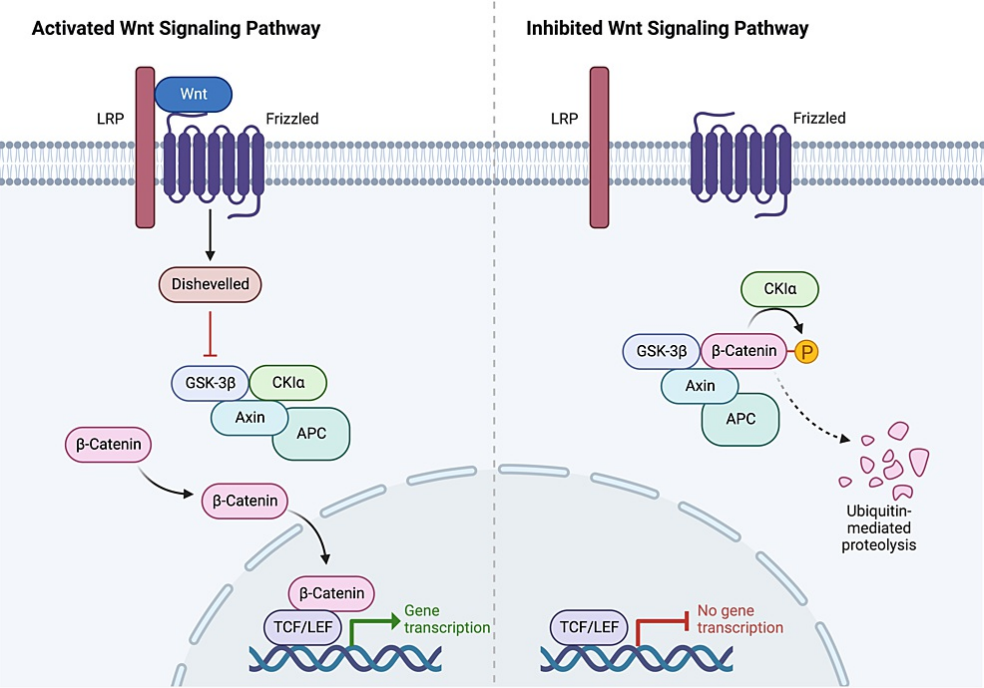
WNT pathway The WNT signaling pathway is a crucial cellular communication pathway involved in development and tissue maintenance. It relies on WNT proteins to transmit signals. Activation occurs when a WNT ligand binds to a receptor, initiating signal transduction and triggering intracellular events. Conversely, inhibition refers to the blocking or suppression of this pathway, regulating its activity and downstream cellular responses. LRP: lipoprotein-related protein, GSK-3β: glycogen synthase kinase-3 beta, CK1α: casein kinase 1 alpha, APC: adenomatous polyposis coli, TCF/LEF: T-cell factor/lymphoid enhancer factor The figure was created by the authors of this article (created with BioRender.com).

The WNT pathway (Figure 1) is one of the main pathways involved in medulloblastoma. WNT-activated MB represents group 1 (10% of cases). This type develops from cells in the dorsal brain stem and lower rhombic lip progenitor cells [15,16]. The majority of WNT-activated MBs exhibit typical morphology, indicative of a low-risk tumor. WNT-activated MB occurrences with a big cell/anaplastic morphology are uncommon. CTNNB1, DDX3X, and TP53 are frequently mutated in WNT-activated MB [17,18].

Sonic hedgehog (SHH)-activated medulloblastoma

**Figure 2:**
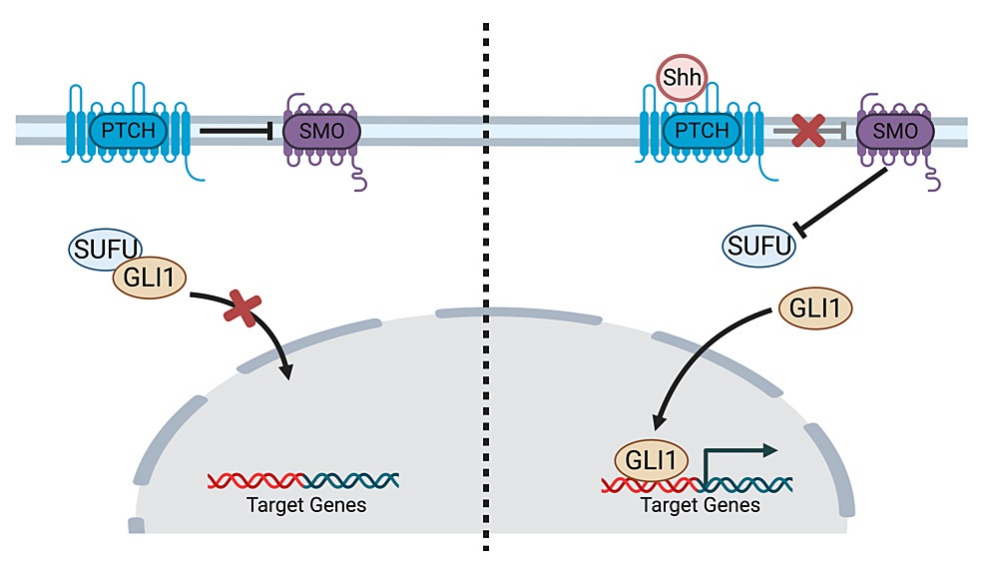
SHH pathway In the absence of Shh, the Ptch inhibits Smo, allowing SUFU to bind and inhibit Gli1. When Shh binds Ptch, Ptch's inhibition of Smo ceases, allowing Smo to inhibit SUFU, freeing up Gli1, which can then enter the nucleus and regulate target gene expression. Shh: sonic hedgehog, Ptch: patched, Smo: smoothened receptor, SUFU: Suppressor of Fused, Gli1: glioblastoma-associated oncogene 1 The figure was created by the authors of this article (created with BioRender.com).

The SHH pathway (Figure 2) is essential for the correct development of the cerebellum. Purkinje neurons release SHH ligands, thus encouraging external granular layer progenitor cells to undergo mitogenesis [19,20]. Of cases of MB, 20% are SHH-activated and can result from several different elements. SHH-activated MB typically manifests in the lateral hemispheres of the cerebellum. The big cell/anaplastic pattern that characterizes TP53-mutant SHH-activated MB may be linked to MYCN amplification, GLI2 amplification, and 17p deletion. The more prevalent type, desmoplastic/nodular TP53-wildtype SHH-activated MB, has been linked to patched 1 (PTCH1) gene and 10q loss [21,22]. Compared to group 3 MB, SHH-activated MB determines metastatic lesions less frequently [20,23,24], but the TP53 variant spreads to the neuroaxis more often.

Group 3 medulloblastoma

MYC amplification, which affects 15%-20% of individuals and is linked to a worse prognosis, is a defining characteristic of group 3 MB. Isochromosome 17q abnormalities are also frequent, as is the activation of GFI1 or GFI1B transcription factors [25]. Approximately 7% of group 3 MBs show amplification of orthodenticle homeobox 2 (OTX-2) [26]. Tetraploidy (17q) is present in 54% of group 3 tumors and is likely an early step in carcinogenesis [27].

Cancer of this type commonly exhibits chromothripsis, which causes odd chromosomal rearrangements or fusions in an unsuccessful effort at DNA repair. Since there are a number of aberrations in the genes involved in this signaling pathway and interactions with subsequent targets like OTX-2, there also seems to be an amplification of transforming growth factor (TGF) signaling [26]. With a survival rate of less than 50% and no survivors over 120 months, this grouping is linked with the poorest prognosis [28].

Group 4 medulloblastoma

With a prevalence of 35%, group 4 is the most frequent molecular subgroup. All ages can be affected, but infants seldom develop it. The male-to-female ratio is 3:1 [26]. In 40% of instances, tetraploidy manifests as an early alteration [27]. MYCN and cyclin-dependent kinase 6 (CDK6) are also often amplified. The majority of females with group 4 tumors lose a copy of the X chromosome, which suggests that at least one tumor suppressor gene may be present on this chromosome [27]. Chromatin modifier changes are typical of group 4 MB, accounting for around 30%-40% of cases. Abnormalities on isochromosome 17q and the activation of GFI1 or GFI1B transcription factors are common in this group, too [25].

Children with WNT MB generally have a good prognosis (>90% survival), while SHH pathway inhibitors exhibit potential in SHH MB [29]; however, the management of young children must be carefully examined due to the chance for premature fusion of growth plates [30,31], and less aggressive treatments are under investigation [32]. Low-risk categories are characterized by hypermethylation, and the high-risk subgroups by hypomethylation. Each grouping is identified through cytogenetic alterations. Telomerase reverse transcriptase (TERT) mutations, large cell anaplastic (LCA) pathology, and MYCN amplification seem to be mutually exclusive. MYCN amplification, TP53 mutation, and LCA pathology are concerning for lower progression-free survival. MYCN amplification and TP53 mutation are also predictors of worse progression-free survival. Chromosome 13 deletion is linked to better results in multivariate analysis, while MYC amplification is a high-risk marker [33].

Current challenges in monitoring and treatment

Medulloblastoma can return, and over 50% of relapses include dissemination. Neuroimaging is usually employed to identify relapse; sometimes, clinical progression occurs first [34]. While useful, imaging-based monitoring has several drawbacks. Imaging methods such as MRI and CT scans offer vital information on morphological changes and tumor development. They could, however, miss some facets of illness development and therapeutic response [34,35]. Imaging is less successful at detecting molecular changes or changes at the cellular level since it primarily records structural changes in tissues. It can be challenging to discern between actual disease progression and pseudo-progression caused by inflammation [36]. Small alterations in size or structure might not be effectively detected, which might result in the treatment response being misinterpreted. Interpretation of imaging results can also be subjective, and variability among different readers or scanners can impact consistency and reliability [37].

Both imaging and CSF cytology demonstrate limitations in sensitivity and specificity when compared to liquid biopsy in detecting spinal cord metastases, irrespective of the metastatic route [2]. When recurrence is discovered radiographically as opposed to clinically, the period to relapse is typically longer [34]. The provision of additional treatment and the assessment of novel approaches in phase I and II trials could both benefit from the detection of asymptomatic recurrence [34]. Parents and children might be more open to taking part in cutting-edge treatments than they would be after the patient starts to show symptoms [34]. As a consequence of persisting, elevated intracranial pressure, ophthalmic problems are common, and vision loss or blindness is possible, particularly in cases of late detection [38]. Although it is usual to perform biopsies for the diagnosis and monitoring of medulloblastoma, this procedure is not devoid of hazards such as infections, bleeding, risks of anesthesia, and pain [39]. This could influence the quality of life and well-being. Scars, for example, can have an impact on self-esteem and body image, especially in children and teenagers. Due to the intrusive nature of the process and the ambiguity surrounding the impending discovery, biopsies can induce emotional anguish and anxiety in patients and families.

The heterogeneity of the disease may not be well reflected by biopsy samples, which can result in errors in diagnosis and therapy formulation [40]. Patients frequently need follow-up for wound care and to keep track of post-procedural signs and symptoms. Survival rates have increased significantly, but medulloblastoma and its therapies have terrible long-term adverse effects that reduce the quality of survival (QoS) [41,42]. Children with tumors of the central nervous system are more vulnerable to neurocognitive impairments and negative social outcomes [43,44]. Hearing loss, subsequent tumor development, neurological and endocrine deficiencies, and other long-term issues could originate from both the malignancy and its therapy [41,42]. Furthermore, surgery may be necessary for radiation-induced cataracts, which can compromise sight. In cases of cranial radiation treatment, lower-than-expected scholastic achievement and a higher demand for specialized school assistance have been recorded [45,46]. Working memory, information processing speed, and sustained concentration can be affected [47,48].

Need for noninvasive biomarkers

The potential to assess a patient's response to therapy, diagnose tumors at earlier stages, assess propagation, carry out periodic surveillance, evaluate tumor volume rather than just the areas that were sampled with a needle, and identify molecular markers for adjuvant therapy could be more significant [49]. Liquid biopsy of medulloblastoma patients analyzed by low-coverage whole genome sequencing (WGS) enabled the detection of molecular alterations early on [50]. They provide information on molecular characteristics at the time of metastatic recurrence when tissue biopsy is not attainable. This improves the targeted therapy possibilities in second- or third-line disease settings. Sources for liquid biopsy include blood, urine, and cerebrospinal fluid. Liquid biopsies work on the principle that tumors shed biological material into the liquid biome, and tumors proliferating at a higher rate are more likely to shed this material. Additionally, liquid biopsies of blood and CSF have been used to identify tumor-specific events such as H3K27M mutations in diffuse intrinsic pontine glioma (DIPG) using droplet digital polymerase chain reaction (ddPCR) [51].

Research demonstrated the utility of low-coverage whole genome sequencing to detect copy number variations as a biomarker of minimal residual disease. Its detection by ctDNA in patients with complete responses preceded disease relapse on imaging by more than three months, which can be life-saving for some patients [50]. In summary, liquid biopsies of CSF in childhood brain tumors could open a route to monitor disease response earlier and more safely and identify molecular targets [50,52]. Creating medulloblastoma representative models of tumor heterogeneity is an important area of preclinical development. Patient-derived cell lines and organoids are a challenge to develop in medulloblastoma compared to other brain tumors, but xenografts are available. Nonetheless, successful engraftment is not guaranteed; there are only about 40% engraft, mostly aggressive types. A study [53] demonstrated that it was the gene expression profiles of a panel of medulloblastoma patient-derived xenografts, rather than mutational aberrations, which best identified target-drug matches. These findings were subsequently validated on patient-derived xenograft lines, although in vitro responses did not consistently match with the predictions. The study also demonstrated that drug prediction analyses can be undertaken on molecular profiles of patients' tumors in conjunction with an in vitro drug screening of tumor cells in vivo. In summary, monitoring the evolution of a tumor in real time allows personalized precision medicine with an immediate clinical response resulting in better outcomes. However, developing models remains a challenge [54].

Liquid biopsies in cancer monitoring

**Figure 3:**
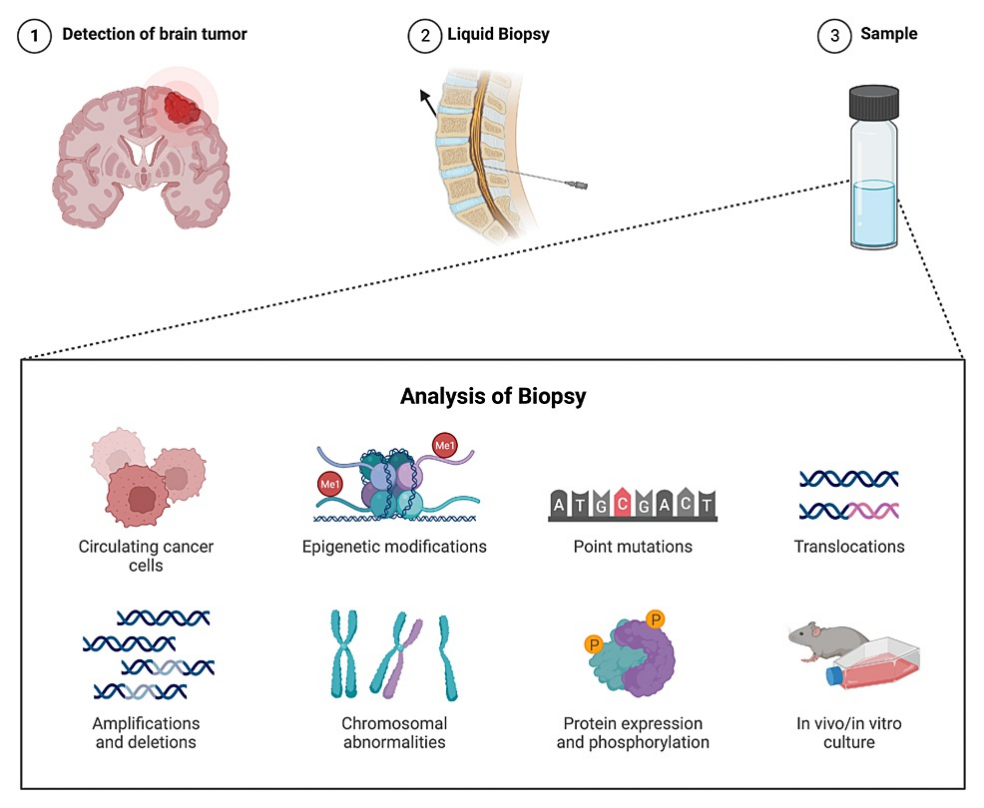
Liquid biopsies Liquid biopsies for brain tumors (1) are preferably performed on CSF (2 and 3). Circulating cancer cells, genetic mutations, epigenetic modifications, chromosomal abnormalities, protein expression, and phosphorylation can be analyzed. In vitro and in vivo cultures can also be prepared and studied. CSF: cerebrospinal fluid The figure was created by the authors of this article (created with BioRender.com).

Different methods have been studied to identify extracellular vesicles (EVs), cell-free nucleic acids (ctDNA/ctRNA), circulating tumor cells (CTCs), and tumor-derived circulating tumor cells (TDCs) in bodily fluids. CSF has emerged as the most suitable option for addressing brain malignancies since it contains a greater amount of markers than blood [55,56]. The emergence of liquid biopsy represents a transformative milestone in the landscape of modern medicine, heralding a paradigm shift in diagnostic approaches [57]. The types of markers typically detectable in liquid biopsies include multiple different biomolecules [58-63]. By analyzing them in liquid biopsies (Figure 3), we can gain a comprehensive and dynamic view of a patient's disease, enabling early detection, accurate diagnosis, treatment monitoring, and disease progression or recurrence assessment. Circulating tumor DNA (ctDNA) is hypothesized to originate from apoptotic and necrotic tumor cells entering the bloodstream [64]. An advantage of ctDNA is its limited association with benign tumors or non-neoplastic diseases, minimizing overdiagnosis [65].

Patients without identifiable circulating tumor cells often exhibit detectable ctDNA, with higher sensitivity seen in metastatic and more advanced cancer stages [58]. ctDNA concentrations can vary among individuals with the same tumor. Some of this variability results from the number of copies of examined genes, with significant variation seen even in non-amplified genes such as TP53 [57]. Decreased ctDNA levels suggest a positive therapeutic response, whereas increased levels may indicate metastatic spread. Conversely, the absence of ctDNA reduction post-treatment could imply poor response, and a subsequent increase after an initial decrease might indicate resistance development. However, interpreting undetectable ctDNA post-surgery as full remission needs careful consideration, as it applies to tumors not directly linked to the cerebrospinal fluid [66].

Recent studies highlight the utility of ctDNA from CSF for MB subgroup identification, risk classification, and monitoring [67], including minimal residual disease (MRD) status prior to radiographic changes [2,4,52]. Furthermore, deep sequencing of CSF ctDNA has been shown to identify more tumor-specific mutations compared to matched tissue, advancing targeted treatment and pharmaceutical investigations [64]. It is essential to recognize the evolving genomic landscape of brain malignancies over time [68,69]. CSF ctDNA collected from individuals with medulloblastoma offers a comprehensive genetic profile accurately representing the specific moment of CSF sampling.

Advantages and limitations of liquid biopsies

Patients are likely to benefit from integrating molecular profiling from solid tumor samples with liquid biopsy, which represents a low-risk method to follow tumor development while offering faster responses than imaging or CSF cytology [2]. In individuals with several metastases under target therapy, it is important to look for early signs of recurrence. Liquid biopsies can quantify the overall tumor burden over time and pinpoint mutations that might develop after treatment [70,71]. While blood has been widely employed for liquid biopsy, its sensitivity for CNS malignancies is low, because of the blood-brain barrier [67]. Furthermore, limited volumes of blood can be safely taken from younger pediatric patients. However, as CSF surrounds the brain and spinal cord, it offers an excellent alternative. In addition, many MB patients have hydrocephalus, which must be treated before surgery. Very few studies have examined the metabolite, lipid, transcriptomic, and genomic patterns in the CSF of children [72,73], despite numerous attempts to identify biomarkers in adults [74].

This is largely because it is technically challenging and is only possible thanks to the development of high-resolution mass spectrometers [75]. CSF carries a small number of RNAs that are highly prone to fragmentation and degradation, and global RNA sequencing of messenger RNAs (mRNAs) and circular RNAs (circRNAs) in CSF presents similar challenges. MB undergoes modifications similar to other cancers: reduced fatty acid oxidation, increased lipogenesis, and a glycolytic phenotype [76]. Tryptophan, methionine, serine, and lysine are upregulated, as well as other hypoxia-induced proteins and metabolites [77]. However, CSF's whole and precise metabolomic architecture in MB has not yet been defined. Standardizing processes to be incorporated into clinical practice is a challenge, albeit it seems easier to do so with ctDNA. CTCs provide a greater challenge for brain tumors, which typically lack the epithelial marker employed to collect carcinomas [78,79].

Imaging frequently does not provide informative data or reflects changes slowly, and patients are exposed to radiation, while ctDNA testing is noninvasive. However, it remains to be established if monitoring patients with advanced illness with any biomarker offers therapeutic advantages beyond psychological ones [58]. Integrative or multivariate data analysis is becoming an important instrument in cancer biology [80]. It can be challenging to gather separate datasets (RNA, protein, lipid, and metabolite) and merge them into one. Liquid biopsies show high specificity but relatively low sensitivity in a diagnostic setting [58,81], but we should not stop at that. For example, liquid biopsy showed greater sensitivity and specificity for diagnosing spinal cord metastasis compared to imaging and CSF cytology [2]. There appears to be a minor benefit of cisternal over lumbar puncture regarding sensitivity when extracting CSF [82].

Individualized genetic analysis of a tumor following the first biopsy can provide several potential markers that may be used in liquid biopsy to subsequently assess the therapy's efficacy. These genetic markers have already achieved a specificity approaching 100% [83,84]. When a rearrangement is found, it takes effort and time to produce and evaluate primers that can effectively find the rearrangement in the degraded DNA. It is easier and less expensive to apply a panel of tests that can identify frequently mutated variants [58]. This might change when liquid biopsies become more common. While CTC identification requires a significant research department, expensive equipment, and funds, ctDNA and mutational analysis seem more in line with clinical practice [2].

Circulating tumor DNA (ctDNA) as a biomarker

The collective pool of cfDNA comprises contributions from various leukocytes and other cell types. While leukocytes dominate this pool, ctDNA stems from apoptotic and necrotic regions of the primary tumor, metastases, and CTCs. Often, ctDNA represents only a minute fraction of the total cfDNA in cancer patients [57]. Technological advancements have yielded approaches for detecting micro-metastases and local relapses even before radiological and clinical indications. These methodologies encompass targeted sequencing of established mutations, non-targeted approaches involving genome-wide analyses, and the innovative field of fragmentomics [85]. It is possible to identify tumor-specific mutations or methylations in ctDNA. We can find several techniques such as digital PCR (dPCR), safe-sequencing system (Safe-SeqS), BEAMing (beads, emulsions, amplification, magnetics), cancer personalized profiling deep sequencing (CAPP-Seq), and tagged-amplicon deep sequencing (Tam-Seq) [85].

Genome-wide approaches for comprehensive profiling

It consists in investigating the entire genome and can be named a non-targeted analysis. It is used when we do not have prior information on specific mutations. Whole exome sequencing (WES), whole genome sequencing (WGS), and the detection of copy number aberrations (CNAs) are some of the techniques. This allows the detection of subclones evolving under treatment or during natural tumor progression. Some of these techniques are able to detect point mutations that were not found in the primary tumor but with specific value for treatment and prognosis [57]. Whole genome sequencing (WGS) is being implemented in clinical environments and provides a comprehensive map of the genetic aberrations that can be used as biomarkers [57]. Long-coverage whole genome sequencing (lcWGS) streamlines experimental workflows and detects new clonal aberrations but misses copy number neutral aberrations or single nucleotide variants (SNVs). It may struggle to detect circulating tumor DNA (ctDNA) in cerebrospinal fluid samples with high cell counts or inflammation, leading to elevated background cell-free DNA (cfDNA). To address these challenges, combining lcWGS and multiplex ddPCR is a promising strategy for future studies [86].

Digital PCR

Integrating structural variant (SV) targets into droplet digital polymerase chain reaction (ddPCR) analysis has the potential to expand the use of this method for tracking tumors with limited single nucleotide variants (SNVs), challenging SNVs, or copy number-neutral SVs. Achieving a turnaround time (TAT) of about 3-4 weeks from receiving whole genome sequencing (WGS) data to having a usable assay allows for sample analysis during and after adjuvant therapy [87].

Fragmentomics

The tumor-derived cfDNA from plasma tends to be shorter in size than normal cfDNA. These differences in fragmentation have been associated with reduced levels of a secreted DNASE1-like nuclease (DNASE1L3) in many tumor types. Future studies are needed to explain the biology of circulating DNA and nucleases to involve fragment size, end motifs, and jagged (single-stranded) ends into clinical applications in tumor biology [57].

Clinical applications of ctDNA in medulloblastoma

Liquid biopsy of ctDNA in CSF showed potential in differentiating between actual progression and pseudo-progression [87], an essential step in treating the heterogeneous disease that is medulloblastoma [67]. Studying ctDNA from CSF offers the advantage of circumventing invasive procedures such as surgical biopsies associated with potential infectious and neurological complications [88]. A lumbar puncture is a straightforward and low-risk method for obtaining CSF samples, making it the optimal source for ctDNA analysis.

Liquid biopsies have demonstrated effectiveness in the monitoring and prediction of disease progression in diffuse midline gliomas (DMGs) and medulloblastomas (MBs) [86]. Genomic analyses have delineated prognostically significant molecular subgroups [89]. There is evidence that highlighted the identification of ctDNA alterations in the CSF of 97.3% of glioma cases [33]. A recent study classified patients into MB subgroups based on common molecular alterations found in the CSF ctDNA WES analysis. The molecular alterations included were PTCH1 and TP53 mutations, SUFU deletion, 17p loss, and MYCN and GLI2 amplification, and facilitated the subgroup classification. Indicators of increased risk include residual disease, metastatic dissemination, LCA histology, or MYCN amplification [31,67]. The quantification of ctDNA holds the potential to signal metastatic progression through an increase in ctDNA levels, while a reduction in ctDNA points toward treatment response. Conversely, the absence of ctDNA reduction following treatment signifies a lack of response, and a subsequent increase after an initial decrease implies the development of resistance [88].

Research established the viability of using ctDNA-based liquid biopsy to monitor medulloblastoma mutations, highlighting that TP53 mutations within sonic hedgehog (SHH)-activated (group 2) subtypes correlate with treatment failures and unfavorable survival rates [57]. Historically, patients with MB were categorized into average-risk or high-risk groups based on residual lesions, metastasis, and histology [88]. A cohort study showed that "sequencing the CSF ctDNA could provide better diagnostic and prognostic information" [67]. The patients were categorized into MB subgroups and risk-stratified [33].

Monitoring disease progression and treatment response

In their classification, the World Health Organization (WHO) has recommended that molecular profiling-based diagnosis surpasses traditional histopathology, particularly for brain tumors [52]. The clinical utility of monitoring therapy response via CSF ctDNA hinges on observing its reduction or escalation. Persistent detection of ctDNA post-treatment or its escalation is associated with an increased risk of progression or metastasis. Conversely, a reduction in ctDNA signals a favorable response to treatment. Notably, a lack of response becomes evident when ctDNA reduction is absent, while a subsequent increase after an initial reduction signifies the development of resistance [52].

In a recent study, molecular profiling was pivotal in tailoring therapy for specific patients. The presence or absence of cfDNA in CSF correlated with various clinical features, including recurrence, metastasis, tumor progression, and the quantity of DNA in CSF [53]. Using epigenetic modifications identified via whole genome methylation sequencing (WGMS) represents an alternative approach for monitoring tumors through multiple CSF samples. DNA methylation and hydroxymethylation profiles within CSF align with signatures from the original tumors in the same patients, enabling the use of ctDNA for monitoring treatment response and tumor recurrence [90].

Real-time assessment of treatment efficacy

The significance of ctDNA as an early detection and diagnostic tool for tumors offers the potential for more timely and potentially curative therapies. This includes the opportunity for treatment intensification in cases of persistent disease or the initiation of second-line or experimental therapies upon relapse [4]. The characteristics of ctDNA facilitate real-time assessment of treatment effectiveness and post-treatment monitoring. Significantly, this approach enhances the quality of life for patients [57]. Furthermore, variations in cfDNA levels were linked to changes in tumor size, pain, and deterioration of condition [57].

Ethical considerations in ctDNA testing

The use of ctDNA poses ethical considerations. Patients need to be fully informed about the purpose, risks and benefits, and limitations of ctDNA testing to make well-informed decisions. In addition, because ctDNA involves analyzing a patient's highly sensitive genetic information, patient privacy and data security should be emphasized [91]. Effective healthcare communication is essential for accurate diagnosis and the development of a successful treatment plan. Patients and their families should receive comprehensive information about various aspects of their condition and treatment. This includes details about therapeutic efficacy, disease staging, potential treatment options, risks and benefits, potential side effects, strategies for managing side effects, expected outcomes, long-term prognosis, and survival rates. Open and clear communication empowers patients to make informed decisions about their healthcare and plays a crucial role in achieving positive treatment outcomes [63].

## Conclusions

In conclusion, this comprehensive review has shed light on the remarkable progress and critical challenges in the diagnosis and monitoring of medulloblastoma. Our exploration has delved into various aspects, from the evolving landscape of liquid biopsies, to the promising role of circulating tumor DNA and miRNAs, to the limitations of current imaging and biopsy-related risks. Liquid biopsies, particularly ctDNA, have emerged as a valuable tool for noninvasive monitoring of medulloblastoma and offer the advantage of early detection, diagnosis, and real-time assessment of treatment response, minimizing the need for invasive procedures. The detection of specific genetic mutations in ctDNA allows for subgroup classification and risk stratification, contributing to personalized treatment strategies. However, challenges persist, including the sensitivity and specificity of ctDNA detection, especially in early-stage disease. The integration of novel technologies, such as next-generation sequencing (NGS) and digital PCR, is poised to enhance ctDNA's diagnostic and prognostic capabilities. While liquid biopsies show immense promise, ongoing research and innovation are essential to harness their full potential. By addressing the current limitations and refining these techniques, we can usher in a new era of improved medulloblastoma management, offering hope and better outcomes for pediatric patients facing this formidable disease.
